# Early Experience With Utilizing Biodegradable Temporizing Matrix (BTM) for Complex Pediatric Wound Reconstruction

**DOI:** 10.1177/22925503261440508

**Published:** 2026-04-20

**Authors:** SK Cho, Y Dhanapala, RL Hartley, AR Harrop, FOG Fraulin, TR Cawthorn

**Affiliations:** 1 Faculty of Medicine, 70401 University of Calgary, Canada; 2 Section of Plastic Surgery, Department of Surgery, 2129 University of Calgary, Canada; 3 Section of Pediatric Surgery, Department of Surgery, University of Calgary, Canada

**Keywords:** Biodegradable Temporizing Matrix, plastic surgery, pediatric, matrice temporisante biodégradable, chirurgie plastique, pédiatrie.

## Abstract

**Background:** Biodegradable Temporizing Matrix (BTM) is a synthetic dermal regeneration template composed of a polyurethane bilayer matrix. Although the use of BTM is well established in adult burn populations, its application for complex pediatric wounds is only beginning to emerge. This case series describes our institution's early experience with BTM for managing a variety of complex pediatric wounds. **Method:** A retrospective chart review was conducted at the Alberta Children's Hospital examining children with complex wounds treated with BTM. Data collected included etiology of the wound, rationale for BTM selection, size of wound, time to wound closure or coverage with split-thickness skin graft (STSG), complications and cosmetic/functional outcomes. **Results:** Eleven children (age range: 2 weeks to 15 years) had wounds treated with BTM between December 2023 and December 2024. The etiology of the wounds varied (eg, trauma, infection, pressure, and postsurgical). Several patients had wound with complicating factors including sepsis, immunosuppression, or exposed critical structures (eg, dura and joint capsule). There was 1 treatment failure in a patient with cognitive delay who removed the BTM 1 week after application. Following BTM application, 6 patients subsequently underwent application of STSG while the other 5 patients healed spontaneously by secondary intention. Wound colonization was the most common complication (5 patients); these were all successfully managed with oral antibiotics. **Conclusion:** BTM is a useful reconstructive option for managing challenging wounds in children and can be utilized in variety of ways. Specific indications for BTM and its relative position on the reconstructive ladder are still evolving. We outline 5 key learning points that may be considered when using BTM in a pediatric population.

## Résumé

**Introduction :** La matrice temporisante biodégradable (BTM) est un substitut dermique synthétique de régénération composé d’une matrice bicouche en polyuréthane. Si son utilisation est bien établie dans la prise en charge des brûlures chez l’adulte, son application aux plaies pédiatriques complexes reste encore émergente. Cette série de cas décrit l’expérience initiale de notre établissement dans l’utilisation de la BTM pour la prise en charge de diverses plaies pédiatriques complexes. **Méthodes :** Une revue rétrospective des dossiers médicaux a été réalisée à l’Alberta Children's Hospital, portant sur des enfants présentant des plaies complexes traitées par BTM. Les données recueillies comprenaient l’étiologie de la plaie, les raisons ayant motivé le choix de la BTM, la taille de la plaie, le délai jusqu’à la fermeture de la plaie ou sa couverture par une greffe de peau mince (split-thickness skin graft, STSG), les complications, ainsi que les résultats esthétiques et fonctionnels. **Résultats :** Onze enfants (âgés de 2 semaines à 15 ans) ont été traités par BTM entre décembre 2023 et décembre 2024. L’étiologie des plaies était variée (notamment traumatique, infectieuse, liée à la pression ou postopératoire). Plusieurs patients présentaient des facteurs de complexité, tels qu’une septicémie, une immunodépression ou l’exposition de structures critiques (par exemple, la dure-mère ou la capsule articulaire). Un échec thérapeutique a été observé chez un patient présentant un retard cognitif, qui a retiré la BTM une semaine après son application. Après la mise en place de la BTM, 6 patients ont secondairement bénéficié d’une greffe cutanée mince (STSG), tandis que les cinq autres ont cicatrisé spontanément par seconde intention. La colonisation de la plaie constituait la complication la plus fréquente (5 patients) ; tous les cas ont été traités avec succès par antibiothérapie orale. **Conclusion :** La BTM constitue une option reconstructive pertinente pour la prise en charge des plaies complexes chez l’enfant et peut être utilisée selon différentes modalités. Les indications spécifiques de la BTM ainsi que sa place dans l’échelle reconstructive restent en cours de définition. Nous présentons cinq points clés susceptibles d’orienter son utilisation en population pédiatrique.

## Introduction

Managing complex soft tissue wounds in children involves unique challenges related to age and developmental status. Skin grafting in young patients is often complicated due to thin skin and relatively limited donor sites due to their size.^1^ Children often have difficulty tolerating the postoperative care that accompanies skin graft donor sites and are prone to inadvertent dressing removal (either intentionally or during activity).^2^ The recent popularization of dermal regeneration templates has provided useful adjuncts for complex wound reconstruction in pediatric patients.

Biodegradable Temporizing Matrix (BTM) is a synthetic dermal regeneration template composed of a polyurethane bilayer matrix.^1^ The bioabsorbable open cell matrix allows for cellular infiltration, while the nonbiodegradable sealing membrane limits moisture loss and acts as a barrier to bacteria.^2,3^ Typically, BTM is used in a staged approach with initial application over the wound followed by subsequent definitive closure several weeks later (once the BTM is vascularized) using a split-thickness skin graft (STSG) over the granulating wound bed.

Although the use of BTM has been well-established in adult burn populations, its use in complex pediatric wounds is only beginning to emerge.^4^ This case series describes our institution's early experience with BTM for managing a variety complex pediatric wounds. These cases have shaped our approach to utilizing BTM in children and we have identified several learning themes from this experience.

## Methodology

All patients with wounds treated using BTM between December 2023 and December 2024 at our institution were identified. Consent was obtained to retrospectively collect patient data and publish deidentified clinical photographs. Data collected included etiology of the wound, rationale for BTM selection, size of wound, time to wound closure or coverage with STSG, complications, and cosmetic/functional outcomes. All cases were collectively reviewed by the clinical team to identify themes and lessons learned from the cases.

Following review by the Conjoint Health Research Ethic Board (CHREB) of the University of Calgary, formal ethics approval was waived due to the nature of the study (retrospective case series).

## Results

Eleven children (age range: 2 weeks to 15 years) had wounds treated with BTM between December 2023 and December 2024 at our institution. All patients were included. Etiology included wounds due to trauma, oncological resection, postoperative dehiscence/nonhealing, infection, pressure injury, and free flap donor sites. The wounds varied in size from 8 to 306 cm^2^. Most patients had associated complicating factors such as sepsis, immunosuppression, active chemotherapy treatment, or exposed critical structures (eg, dura and joint capsule).

The BTM was typically secured to the peri-wound using sutures or staples. A nonadherent contact layer (ie, bismuth-impregnated fine gauze and nonadherent silicone dressings) was applied directly over the BTM sealing membrane. Depending on the anatomic location, the dressing was then secured using either a gauze and elastic wrap or negative pressure wound therapy. In this case series, 6 of the 11 cases utilized negative pressure wound therapy over the BTM.

Following BTM application, 5 patients subsequently underwent application of STSG (median 35 days after BTM application) while the other 5 patients healed by secondary intention (median 89 days after BTM application). There was one treatment failure in a patient with cognitive deficits who removed the BTM 1 week after application. Wound colonization with *Staphylococcus aureus* was the most common complication (n = 5); these cases were successfully managed with antibiotics. Summary of the cases is shown in Table 1 and specific details of each case are as described below.

**Table 1:** Summary of the Cases.

**Case number**	Age at BTM application	Etiology	Location	Rationale for using BTM	Size (cm^2^)	Skin graft utilized?	Time to skin graft after BTM application (days)	Time to healing by secondary intention (days)	Complications
1	3 years	Wound dehiscence	Lumbar spine	Exposed dura in setting of ongoing chemotherapy. Immunosuppresed	15	No	n/a	88	None
2	2 weeks	Area of full thickness scalp necrosis (multifactorial—sepsis, prematurity, pressure, vasopressors)	Occipital scalp	Temporizing solution for a neonate too ill for definitive closure	38	No	n/a	93	*Staphylococcus aureus* colonization
3	10 years	Skin necrosis around previous sarcoma resection and flap reconstruction	Left proximal tibia	Uncertain wound bed in patient on chemotherapy. Immunosuppressed	10 and 21	Yes	29	n/a	None
4	10 months	Necrotizing soft tissue infection around G-tube site	Abdomen	Temporizing solution for massive defect in infant with inadequate donor sites for skin grafting	228	No	n/a	179	*Streptococcus agalactiae, S. aureus* infection Scar contracture
5	7 years	Full thickness traumatic skin defect	Medial aspect of foot	Exposed joint capsule and bone	100	No	n/a	76	Wound infection with MRSA
6	10 years	Plexiform fibrohistiocytic tumor	Left anterior shoulder	Awaiting final pathology to ensure negative margins prior to reconstruction	100	Yes	26	n/a	MSSA and group G strep wound infection
7	9 years	Retiform hemangioendothelioma	Right plantar foot	Awaiting final pathology to ensure negative margins prior to reconstruction	25	Yes	42	n/a	*S. aureus* colonization
8	15 years	Pressure sore	Right posterior heel	Wound be with exposed Achilles tendon and calcaneus	8	No	n/a	181	Treatment failure. Patient removed dressings and BTM
9	15 years	Skin necrosis after above knee amputation	Stump site	Large defect with contour irregularities	306	Yes	34	n/a	*S. aureus* colonization
10	9 years	Skin necrosis from motor vehicle injury wounds and around pin for external fixator	Left lateral aspect of foot overlying lateral malleolus	Wound with exposed lateral malleolus ligaments	38	Yes	35	n/a	*S. aureus* colonization
11	13 years	Radial forearm free flap donor site	Volar aspect of distal forearm	Wound with contour irregularities	56	Yes	39	n/a	

Abbreviation: BTM, Biodegradable Temporizing Matrix; MRSA, methicillin-resistant *Staphylococcus aureus*; MSSA, methicillin-sensitive *Staphylococcus aureus*.

### Case 1: 3-Year-Old Boy With a Spine Wound Dehiscence on Active Chemotherapy

**Figure 1.**
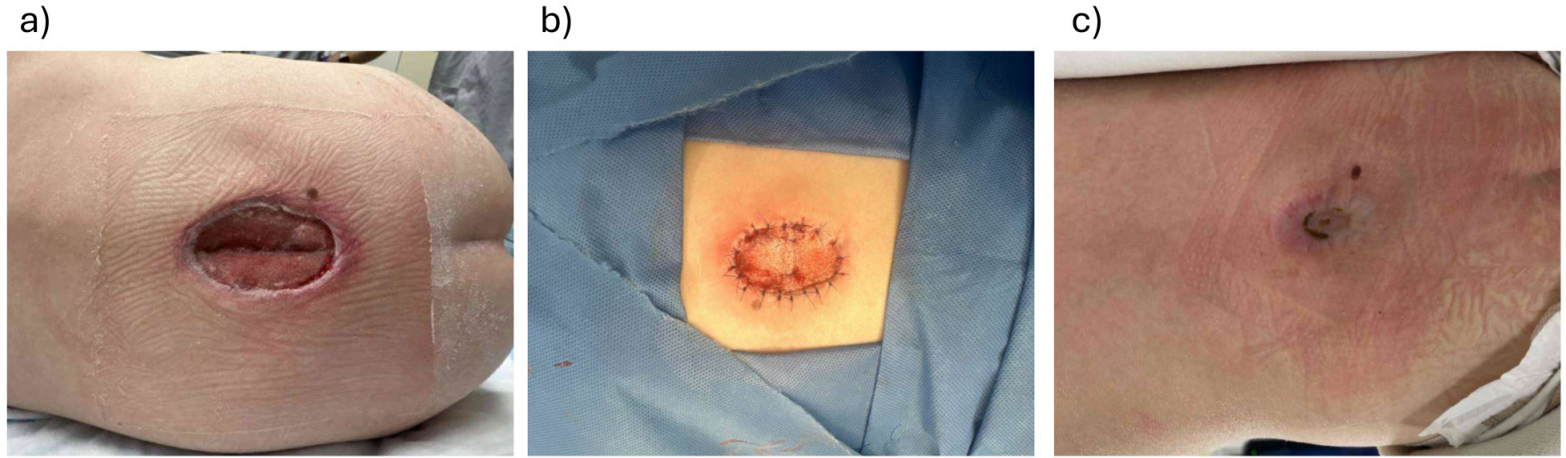
(a) A 3-year-old with wound dehiscence to previous surgical scar; (b) at the time of Biodegradable Temporizing Matrix (BTM) application; and (c) 3 months post-BTM application.

A 3-year-old boy with medulloblastoma underwent an L5-S1 laminectomy for spinal cord decompression and to biopsy a metastatic lesion (Figure 1). The patient was being concurrently treated with high-dose chemotherapy and postoperatively developed a 5 cm × 3 cm wound dehiscence with exposed muscle and dura. The wound did not respond to negative-pressure therapy and primary closure was not possible due to the wound size and location.

BTM was chosen for reconstruction due to the poor wound bed (exposed dura) and concerns for impaired wound healing in the context of active chemotherapy. BTM was applied to the wound 2 months after the original laminectomy procedure. Chemotherapy proceeded as planned and 3 months after BTM application the wound was fully re-epithelialized; skin grafting was not required.

### Case 2: Premature Infant With a Scalp Wound and Severe Sepsis

**Figure 2.**
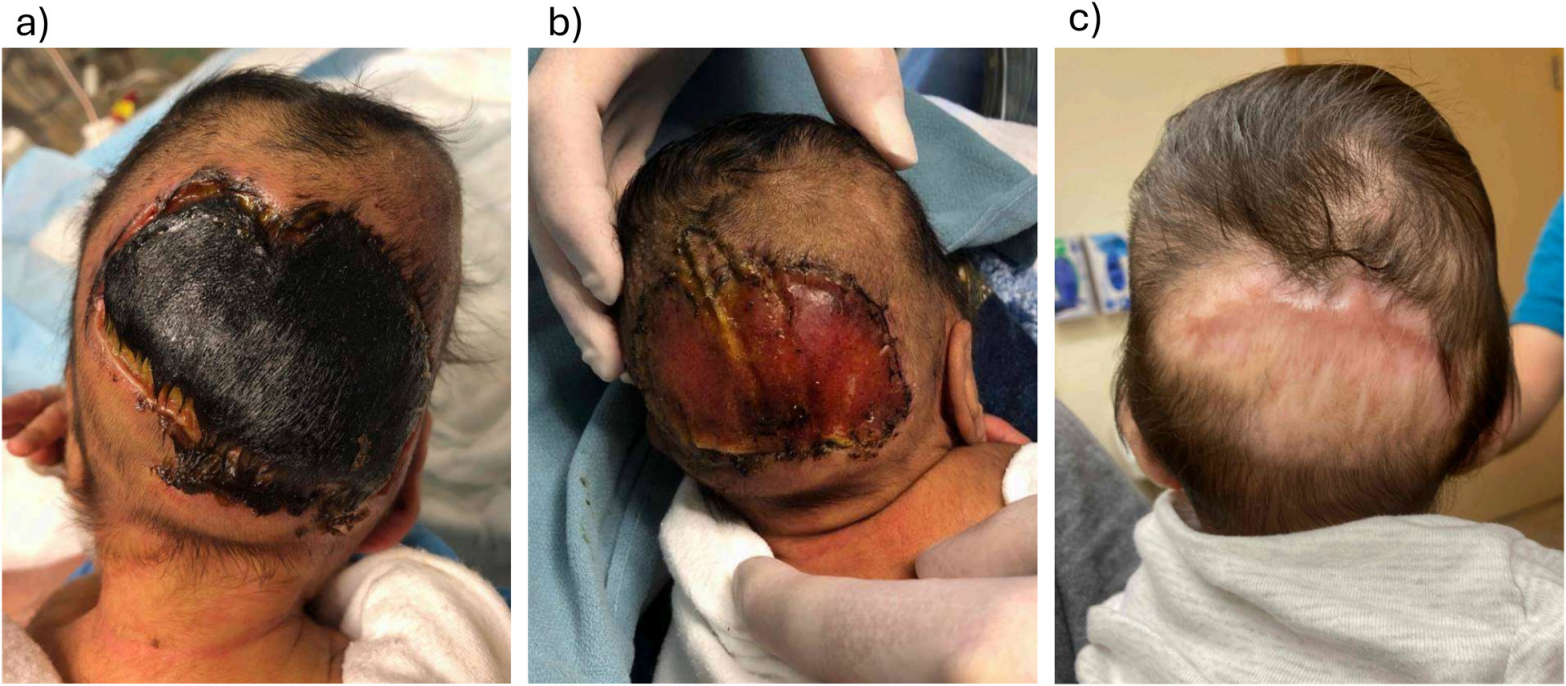
(a) A 2-week-old with full thickness scalp necrosis; (b) at the time of Biodegradable Temporizing Matrix (BTM) Application; and (c) 8 months post-BTM application.

A 2-day-old boy, delivered at 32 weeks gestation, was admitted to the neonatal intensive care unit for sepsis requiring high-dose vasoactive support (Figure 2). Examination revealed a 7.5 cm × 5 cm area of full-thickness skin necrosis involving the occipital scalp. The working diagnosis was an iatrogenic pressure injury from supine positioning that was exacerbated by vasopressor use.

BTM was selected for wound management due to the patient's age, limited available donor sites for skin grafting, and being too ill for definitive closure. At 2 weeks of age, the wound was debrided to periosteum and BTM was applied. Although the original intention was for subsequent skin grafting, the wound healed by secondary intention 10 weeks after BTM application, obviating the need for grafting.

### Case 3: 10-Year-Old Girl With a Postsurgical Leg Wound on Active Chemotherapy

**Figure 3.**
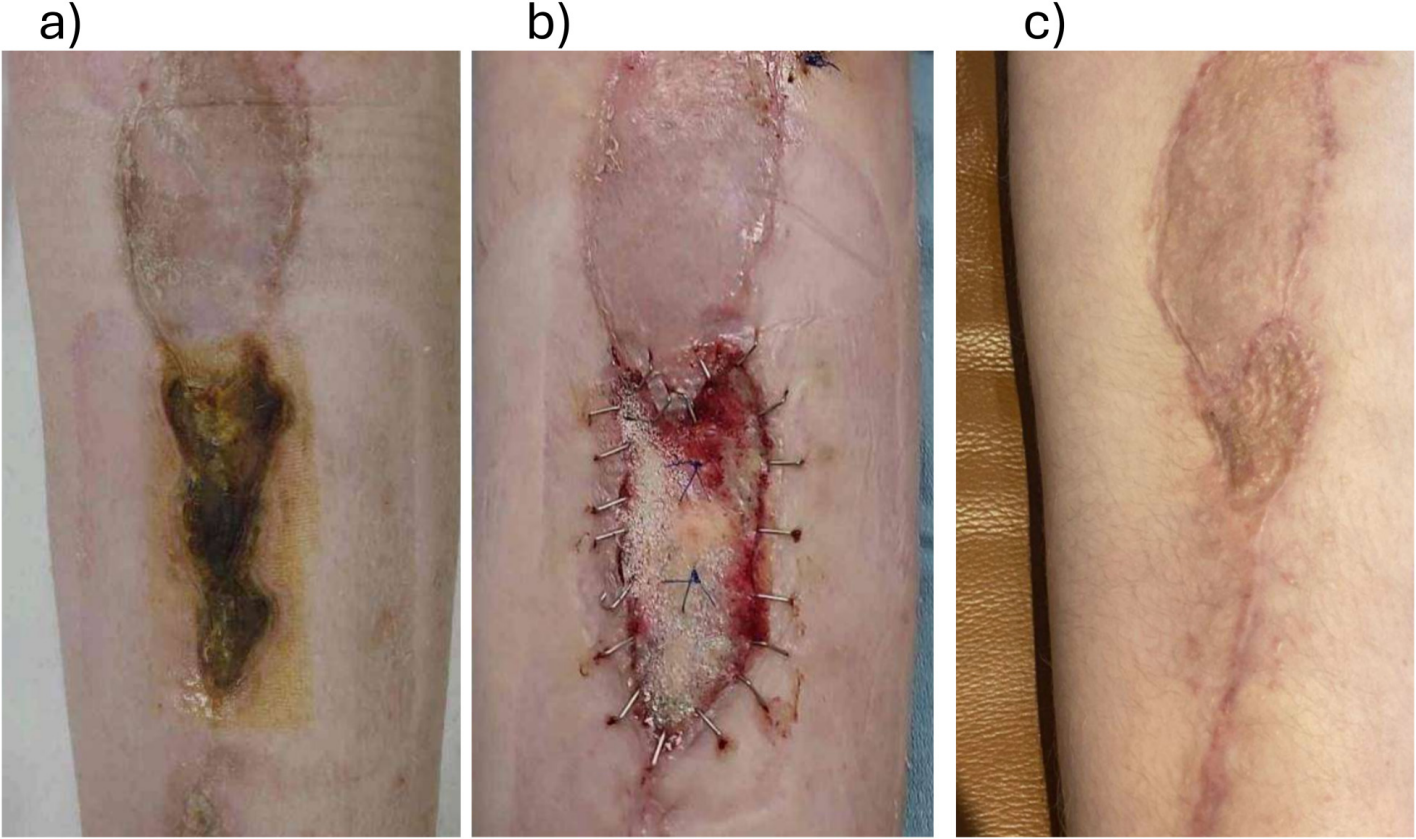
(a) A 10-year-old with skin necrosis around previous sarcoma resection; (b) at the time of Biodegradable Temporizing Matrix (BTM) application; and (c) 4.5 months post-BTM application, 3.5 months post-split-thickness skin graft application.

A 10-year-old girl underwent resection of a left proximal tibial sarcoma and reconstruction with a contralateral free fibula flap (Figure 3). Twelve days postoperation, there was marginal skin necrosis at the incision. The necrosis was monitored while she completed 5 months of chemotherapy. Once chemotherapy was completed, BTM was chosen to reconstruct the wound base due to a nongranulating, fibrinous wound bed that was not suitable for skin grafting. One-month post-BTM application, definitive closure over the BTM was completed with an STSG.

### Case 4: 10-Month-Old Boy With a Large Abdominal Wound and Limited Skin Graft Donor Sites

**Figure 4.**
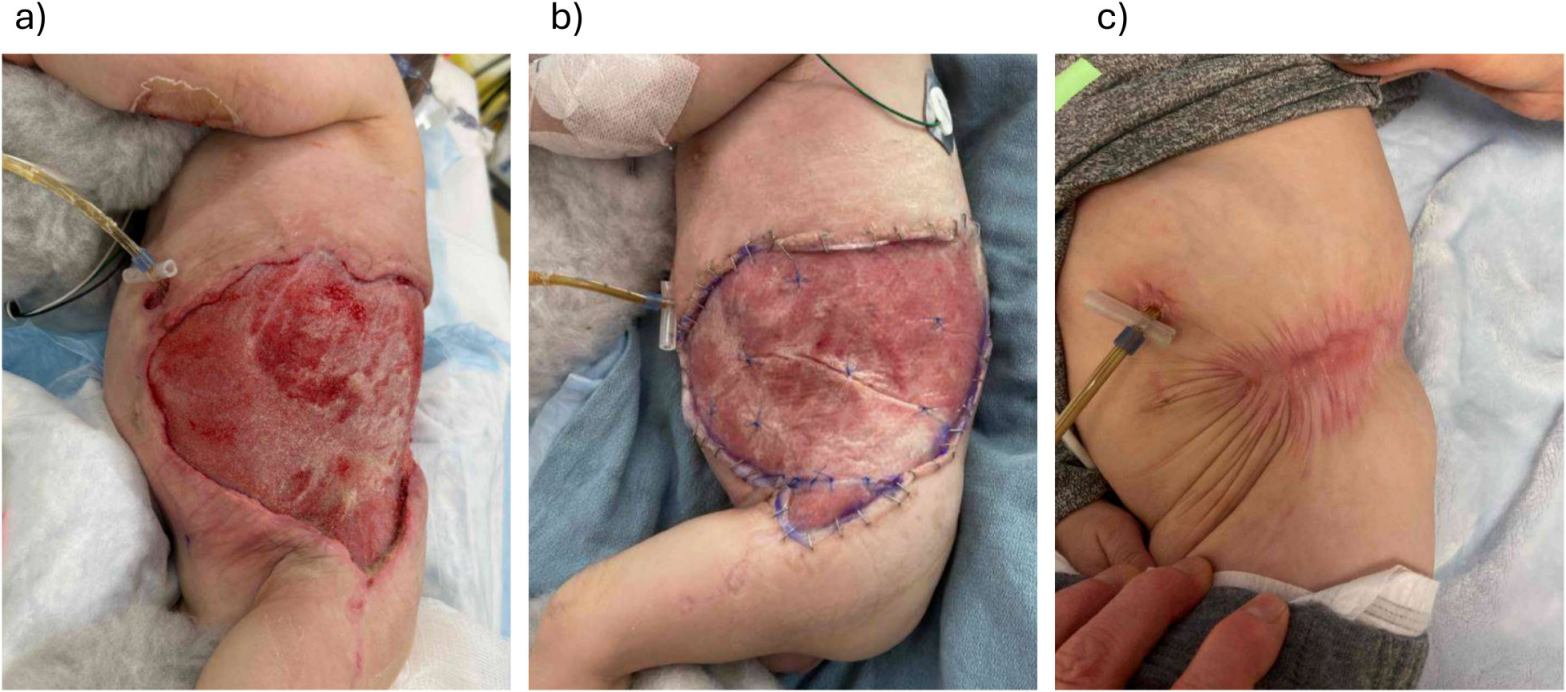
(a) A 10-month-old with necrotizing soft tissue infection around G-tube site; (b) at the time of Biodegradable Temporizing Matrix (BTM) application; and (c) 7 months post-BTM application.

A 10-month-old boy with Costello syndrome was admitted to the pediatric intensive care unit with septic shock and necrotizing cellulitis around a recent G-tube insertion site (Figure 4). He required multiple surgical debridements and the resultant wound measured 12 × 19 cm with exposed abdominal wall fascia. BTM was selected for reconstruction due to the size of the defect, limited available donor sites, and poor nutritional status of the patient.

Following BTM application, he had several episodes of suspected wound infection which were managed with oral antibiotics. However, over the course of 6 months, the wound contracted and completely re-epithelialized without the need for skin grafting. He has developed some scar contracture across the hip area which is being managed with scar massage, stretching, and silicone products.

### Case 5: 7-Year-Old Boy With a Traumatic Foot Wound With Exposed Joint Capsule and Bone

**Figure 5.**
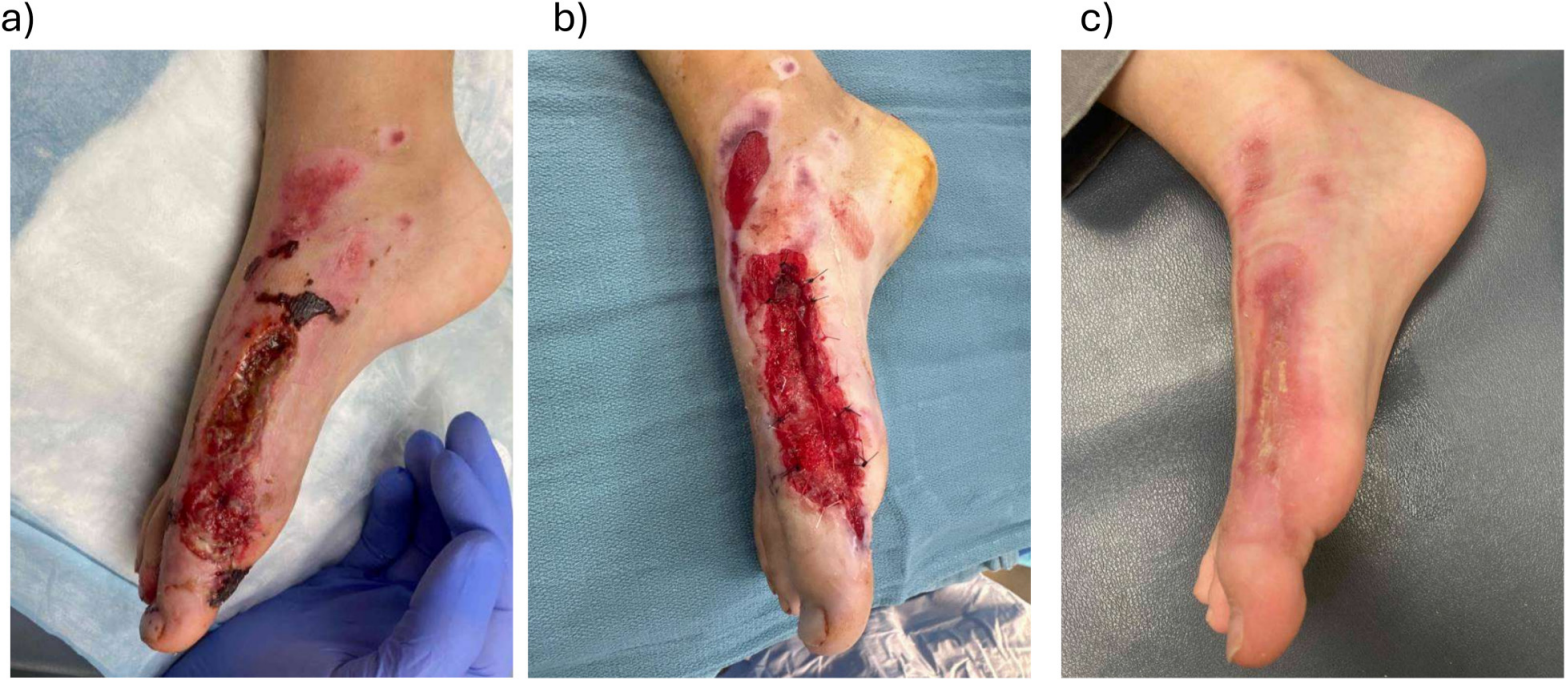
(a) A 7-year-old with full-thickness traumatic skin defect; (b) at the time of Biodegradable Temporizing Matrix (BTM) application; and (c) 3 months post-BTM application.

A 7-year-old boy sustained a right first metatarsal open fracture with extensive soft tissue abrasions from riding a motorized scooter while traveling internationally (Figure 5). He was initially treated with debridement and K-wire fixation in another country. Upon return to Canada, he had a wound on the dorsal/lateral foot with exposed extensor tendon, bone, and joint capsule. Due to the exposed structures, the wound was not suitable for skin grafting and BTM was applied after further debridement.

Two weeks post-BTM application, the patient developed cellulitis around the wound which was managed with antibiotics and the BTM was salvaged. Ten weeks after BTM application, the wound had healed by epithelialization and did not require skin grafting. The healed area demonstrated good integrity and did not impact range of motion.

### Case 6: 10-Year-Old Boy With a Left Shoulder Oncological Resection Wound Awaiting Final Pathology

A 10-year-old male underwent resection of a plexiform fibrohistiocytic tumor, a rare tumor of mesenchymal origin. Due to positive margins on final pathology, the patient required re-excision, resulting in a wound that could not be closed primarily. BTM was applied to temporize the wound while awaiting pathology results to ensure negative margins prior to proceeding with definitive reconstruction.

Two weeks post-BTM application, the wound became infected with methicillin-susceptible *Staphylococcus aureus* and *Group G Streptococcus*; he was subsequently treated with oral antibiotics. The BTM remained intact and adherent to the wound bed. One month after BTM application, an STSG was applied for definitive wound closure.

### Case 7: 9-Year-Old Boy With a Right Foot Resection Wound Awaiting Final Pathology

A 9-year-old male with IPEX syndrome (immune dysregulation polyendocrinopathy, enteropathy, X-linked syndrome) presented with a right plantar foot retiform hemangioendothelioma, a rare intermediate grade vascular tumor which commonly recurs but rarely metastasizes. Consequently, a wide excision (5 cm × 5 cm, including plantar fascia) was completed. Immediately following resection, BTM was applied to temporize the wound while awaiting final pathology to ensure clear margins prior to definitive reconstruction. Following confirmation of negative margins, the BTM was resurfaced using an STSG 6 weeks later.

### Case 8: 15-Year-Old Female with a Right Heel Pressure Wound

A 15-year-old female with trisomy 21 and severe cognitive delay developed a 5 × 4 cm full-thickness pressure sore to the right posterior heel following 4 weeks of casting after a right knee orthopedic operation. The wound was debrided, revealing an ungraftable wound with exposed Achilles tendon and calcaneus periosteum. Due to the poor wound bed, BTM was utilized for reconstruction. Unfortunately, shortly after the application of BTM, the patient removed the dressings and the BTM itself. Due to concerns with patient compliance, the wound was subsequently treated with dressings and eventually healed by secondary intention approximately 5 months later.

### Case 9: 15-Year-Old Boy With Tissue Necrosis at Above Knee Amputation Site

A 15-year-old male suffered a severe crush injury to his left leg that required an above knee amputation. Unfortunately, the amputation stump subsequently developed a large area of tissue necrosis. Following debridement, the resultant skin defect measured 17 cm × 18 cm. BTM was chosen for reconstruction due to the size of the defect and to help with the contour irregularities in the area in preparation for skin grafting. Approximately 5 weeks after BTM application, an STSG was applied over the defect. The final reconstruction was durable and suitable for use of a prosthesis without wound breakdown.

### Case 10: 9-Year-Old Boy With Traumatic Left Foot Wounds

A 9-year-old male sustained extensive injuries from a motor vehicle collision including a left foot fracture-dislocation which required management with an external fixator. Skin necrosis developed along the lateral aspect of the foot overlying the lateral malleolus around one of the external fixator pins. The area required debridement and the resultant full thickness defect measured 7.5 × 5 cm with exposure of the lateral malleolus ligaments. BTM was used to cover the exposed ligamentous structures. Approximately 5 weeks after BTM application, an STSG was applied to complete the reconstruction.

### Case 11: 13-Year-Old Girl With Radial Forearm Free Flap Donor Site

A 13-year-old girl underwent resection of a right ankle fibromyxoid sarcoma and immediate reconstruction with a left radial forearm free flap. Due to the obvious contour defect at the forearm donor site, BTM was applied immediately following the flap harvest. Just over 5 weeks later, she underwent STSG over the BTM. At this operation, a significant improvement in forearm contour was noted with excellent vascularization of the BTM. The resultant skin graft went on to heal without any complications.

## Discussion

This case series represents our early experience with utilizing BTM for complex pediatric wounds. The 11 cases presented highlight the versatility of BTM in managing wounds of different etiologies and with various complicating factors. Although the literature on BTM for wound reconstruction is well-established in the adult population, there is a relative paucity of reports in children.^4,5^ We have identified 5 key learning points that may be considered when using BTM.

### BTM Can Convert Nongraftable Wounds to Graftable Wounds

The primary indication for utilizing BTM is to convert poor wounds beds into healthy, granulating wounds which can receive a skin graft. Our experience confirms this capability: BTM was successfully applied to wounds that would traditionally require complex reconstruction techniques due to their size, location, and/or wound bed. Several of these wounds may have necessitated free tissue transfer, but the use of BTM obviated the need for such complicated operations. This subsequently permitted wound closure using much more straightforward approaches (ie, skin grafting or healing by secondary intention) with shorter operative times and less complex postoperative courses.

Avoiding long and complex operations has a multitude of potential benefits, both from a patient perspective and system perspective. Patients with pre-existing medical complexity or comorbidities may have difficulty tolerating the prolonged general anesthetics that typically accompany free tissue transfer procedures. Reconstruction with BTM followed by skin grafting does require 2 operations, but both procedures are typically short duration (less than 1 h). Furthermore, with this approach patients can often be managed on an outpatient basis and avoid prolonged hospital admissions. Once the BTM has been applied, dressing changes can be completed on a weekly basis until the BTM has fully vascularized and is ready for definitive grafting.

### BTM May be Used to Temporize Wounds

BTM application provides an opportunity to safely temporize open wounds prior to definitive reconstruction. Once the BTM has been applied, the wound may be considered “closed” via the polyurethane membrane and requires a minimum of several weeks for revascularization. Closure of the wound reduces the patient's metabolic demand during the revascularization period.

In our case series, we identified several potential scenarios where BTM was used in a temporizing fashion:

1. Patients with comorbidities precluding immediate reconstruction. The youngest patient in our series (preterm infant, 2 weeks of age) exemplifies the benefits of this approach. He had a large occipital scalp pressure wound but was also systemically unwell and requiring intravenous antibiotics. Debridement and application of BTM was a short and simple procedure which enabled immediate wound closure (without a donor site) while he recovered from his systemic illness.
2. Patients on active chemotherapy impacting wound healing potential. Chemotherapy inhibits normal healing mechanisms and can lead to chronic, nonhealing wounds. In these cases, BTM can be applied to the wound for temporary closure until the course of chemotherapy is completed. This allows the wound to be “closed” during this time, reducing metabolic demand and risk of infection. Once chemotherapy is completed, the patient can then undergo definitive reconstruction.
3. Patients awaiting final pathology results after undergoing oncological resection. Patients with locally aggressive cutaneous tumors may benefit from utilizing BTM to temporize the wound prior to definitive reconstruction. This ensures that the final margins are negative before a skin graft or more complex reconstruction is performed. This approach provides a simple initial reconstructive solution that minimizes the risk of requiring multiple reconstructions if resection margins are positive.

### Wounds Resurfaced With BTM May Spontaneously Re-Epithelialize

Although BTM is designed to be utilized in a staged fashion (application and subsequent vascularization, followed by STSG), reports have described complete re-epithelialization over the BTM without grafting.^25,26^ Due to a variety of factors, several of our patients were not candidates for skin grafting in the weeks after BTM application. We observed that many of these patients’ wounds spontaneously epithelialized over the vascularized BTM. During this process, the sealing polyurethane membrane gently lifted off as the wound closed from the margins.

In our case series, 4 patients had wounds resurfaced with BTM that ultimately did not require skin grafting. Although spontaneous wound closure over BTM does take longer than resurfacing with a skin graft, this approach can be helpful in specific situations. Several of our patients were medically unwell and not fit for multiple operations or were felt to have difficulty tolerating a large skin graft donor site. In similar situations, it may be reasonable to consider delaying the subsequent skin grafting procedure as the wound size may be reduced or completely close in the interim. Further research is required to determine the long-term implications of allowing wounds to re-epithelialize over vascularized BTM.

### BTM is Tolerant of Bacterial Colonization

BTM appears to be more resistant to colonization and infection than other dermal substitutes given its synthetic nature.^6^ Reports have indicated lower rates of infection of BTM compared with Integra^®^ Dermal Regeneration Template, another commonly used skin substitute made from bovine tendon collagen and shark glycosaminoglycans.^2,7^ In our series, 7 patients had positive wound swabs post-BTM application, with *Staphylococcus aureus* being the most common organism. Despite this, none of the patients required debridement or replacement of the BTM. The BTM remained well adhered during treatment with antibiotics, and the reconstruction was salvaged in all cases.

Although BTM appeared to tolerate bacterial colonization in our case series, the number of cases was limited. A recent systematic review found that that infected wounds had lower BTM survival than noninfected wounds.^8^ However, the review noted several reports of acceptable BTM adherence and skin graft survival despite infection.^9–15^ These finding highlight that clinical equipoise still exists with respect to management of wounds treated with BTM that are found to have bacterial colonization or overt infection.

### BTM May Improve Contours for Final Reconstruction

An STSG will mold to the shape of the wound bed it is applied to. Unfortunately, this can lead to unfavorable aesthetic results in wounds with abnormal or unusual contours. However, we have found that BTM has the capacity to improve wound contour abnormalities. Contour changes may occur throughout the BTM vascularization process as new granulation tissue forms within, and superficial to, the foam matrix. Although our case series did not specifically address this question, we anecdotally found a significant improvement in overall wound contour following BTM application.

We have begun to consider using BTM on certain wounds with unusual contours *even if the wound bed is graftable*. For example, in one case, BTM was applied to a radial forearm free flap donor site with the intention of improving this (typically) unaesthetic site. We were very encouraged by the change in contour as the BTM vascularized and granulated. Consequently, this is now our preferred approach for managing this donor site (and others with poor contours).

## Conclusions

Our early experience confirms that BTM is a versatile reconstructive tool for managing challenging wounds in a pediatric population. It can be utilized in a variety of clinical settings and provides surgeons with a reliable option for wound closure that does not require a donor site. We have found that the temporizing ability of BTM is particularly useful in situations where patients may not be candidates for immediate reconstruction. Despite this positive early experience, further work is required to refine the specific indications for BTM in managing pediatric wounds.
